# Accurate Acetabular Component Positioning in Total Hip Arthroplasty Using Preoperative CT and Anatomical Landmarks

**DOI:** 10.3390/jcm15176766

**Published:** 2026-08-31

**Authors:** Min Uk Do, Kyeong Baek Kim, Sang-Min Lee, Hyun Tae Koo, Kuen Tak Suh, Won Chul Shin

**Affiliations:** 1 Department of Orthopedic Surgery, Research Institute for Convergence of Biomedical Science and Technology, Pusan National University Yangsan Hospital, Pusan National University School of Medicine, Yangsan 50612, Republic of Korea; do7613@naver.com (M.U.D.); pnuyh.102@gmail.com (K.B.K.); dh62121@gmail.com (S.-M.L.); sta9711@naver.com (H.T.K.); 2 Department of Orthopedic Surgery, Mirae Hospital, Pusan National University School of Medicine, Busan 47889, Republic of Korea; kuentak@pusan.ac.kr

**Keywords:** total hip arthroplasty, anteversion, computed tomography, anatomical landmark, acetabular component

## Abstract

**Background:** Accurate acetabular component anteversion during total hip arthroplasty (THA) remains challenging. We evaluated a preoperative computed tomography (CT)–based technique that uses patient-specific acetabular bony landmarks to position the cup. **Methods:** We prospectively enrolled 48 patients (50 hips) undergoing primary THA between September 2023 and January 2024. On preoperative CT, the anterior acetabular notch (AAN) was defined as the anterior pole, and the point opposite it across the acetabular center was defined as the posterior pole. Using the cup radius (r), the target-relative rotation (θ), and half the interpolar distance (R), the arc-length distances from each pole to the cup margin (a and b) were derived preoperatively with exact circular-geometry equations and reproduced intraoperatively. Postoperative CT anteversion was measured and converted to radiographic anteversion to determine outliers from the Lewinnek safe zone. Early clinical outcomes, including dislocation and modified Harris hip score (mHHS), were assessed at 3 months. **Results:** Mean postoperative CT anteversion was 25.1° (range, 16.0–35.0°), with a mean absolute error of 4.0° (range, 0–12.0°). Calculated radiographic anteversion was 16.8° (range, 10.0–24.0°), and all 50 hips were within the Lewinnek safe zone. At 3 months, mean mHHS was 92.4, and no dislocations were observed. **Conclusions:** This prospective proof-of-concept study suggests that CT-based acetabular bony landmarks can enable accurate cup positioning in standard primary THA. Further controlled studies are required to confirm reproducibility and clinical advantage.

## 1. Introduction

Setting accurate anteversion of the acetabular component in total hip arthroplasty (THA) remains challenging [1,2,3,4,5]. Malposition of the acetabular component outside this target has been reported in a substantial proportion of cases, even in experienced hands. This is largely because, with freehand placement, the surgeon must visually estimate version under intraoperatively variable pelvic positioning, and even small changes in pelvic tilt can directly alter functional cup orientation [6]. The Lewinnek safe zone, defined as a radiographic inclination of 40° ± 10° and a radiographic anteversion of 15° ± 10°, has historically been used to define acceptable cup orientation, and placement outside this range has been associated with higher rates of dislocation, edge loading, and revision [3].

These difficulties have motivated a range of solutions, including computer navigation, robotic assistance, and intraoperative anatomical references. Although robotic surgery has been introduced to improve the precision of acetabular cup positioning, several drawbacks remain, including cost and a learning curve of 12–35 cases [7,8,9]. Anatomical landmark-based techniques offer a simpler alternative that can be applied directly within the surgical field. The transverse acetabular ligament (TAL), introduced by Archbold et al. [10], has been widely used to guide acetabular cup positioning, and excellent results have been reported [11,12]. However, identification and reliable use of the TAL may be difficult, particularly during the learning curve or in cases where the ligament is not clearly visualized [13].

In addition to soft-tissue landmarks such as the TAL, fixed bony structures can also serve as intraoperative anatomical references [14]. Recent work has suggested that bony landmarks, including the anterior and posterior acetabular notches, may provide constant and reproducible references for cup orientation that are relatively independent of pelvic position [15], and that landmark-based guidance can achieve target orientation more consistently than freehand technique [16]. If an appropriate reference point is selected, bony landmarks have the advantages of being readily identifiable and less affected by patient positioning. Therefore, we propose a new technique for positioning the acetabular cup using acetabular bony landmarks.

**Figure 1:**
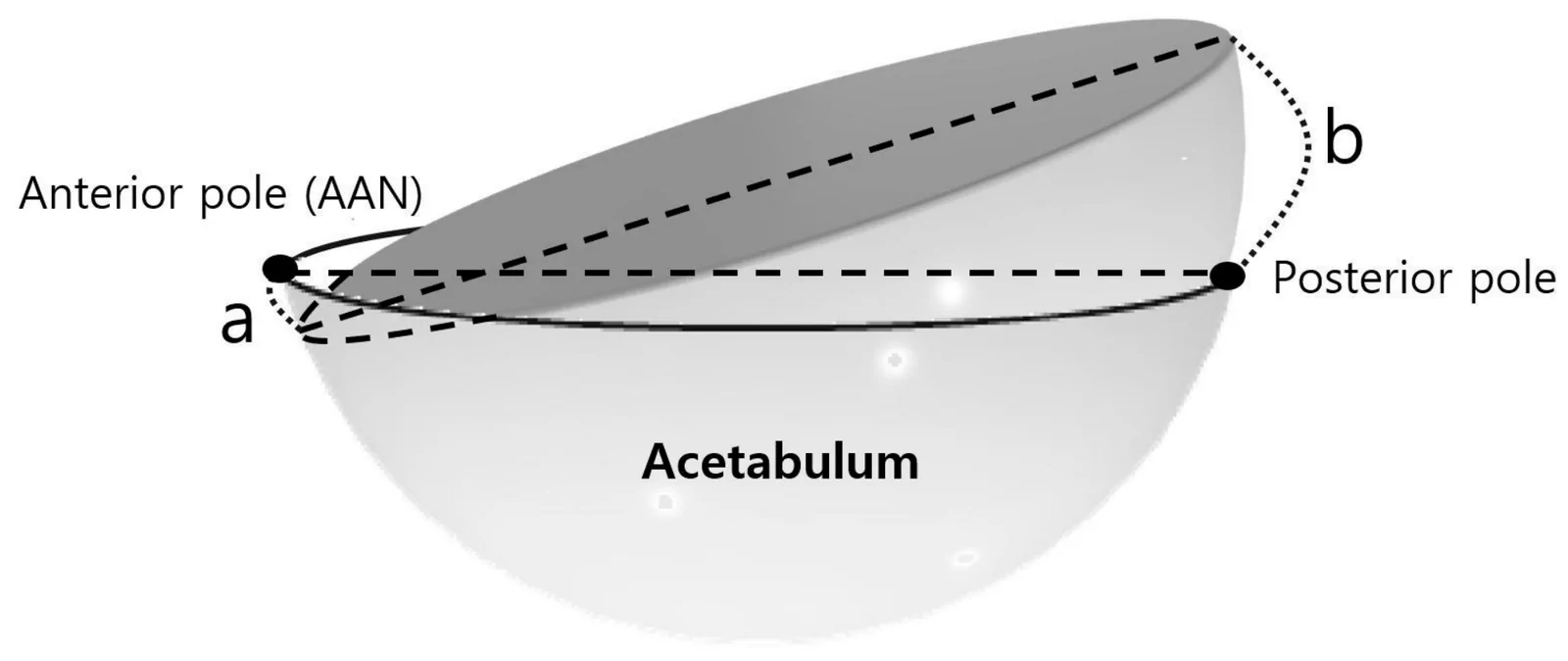
Schematic of positioning the acetabular cup in the acetabulum. a and b represent the arc lengths measured along the cup rim from the anterior and posterior acetabular poles, respectively, to the cup margin.

We hypothesized that if the anterior and posterior poles of the acetabulum where the acetabular cup is to be positioned are known, the cup could be accurately placed in the desired location by determining the distance between the cup and the poles (Figure 1). Computed tomography (CT) was used to identify the anterior and posterior poles of the acetabulum. By calculating the distances between the acetabular component and the poles preoperatively, we aimed to position the cup accurately.

## 2. Materials and Methods

### 2.1. Study Design and Patients

**Figure 2:**
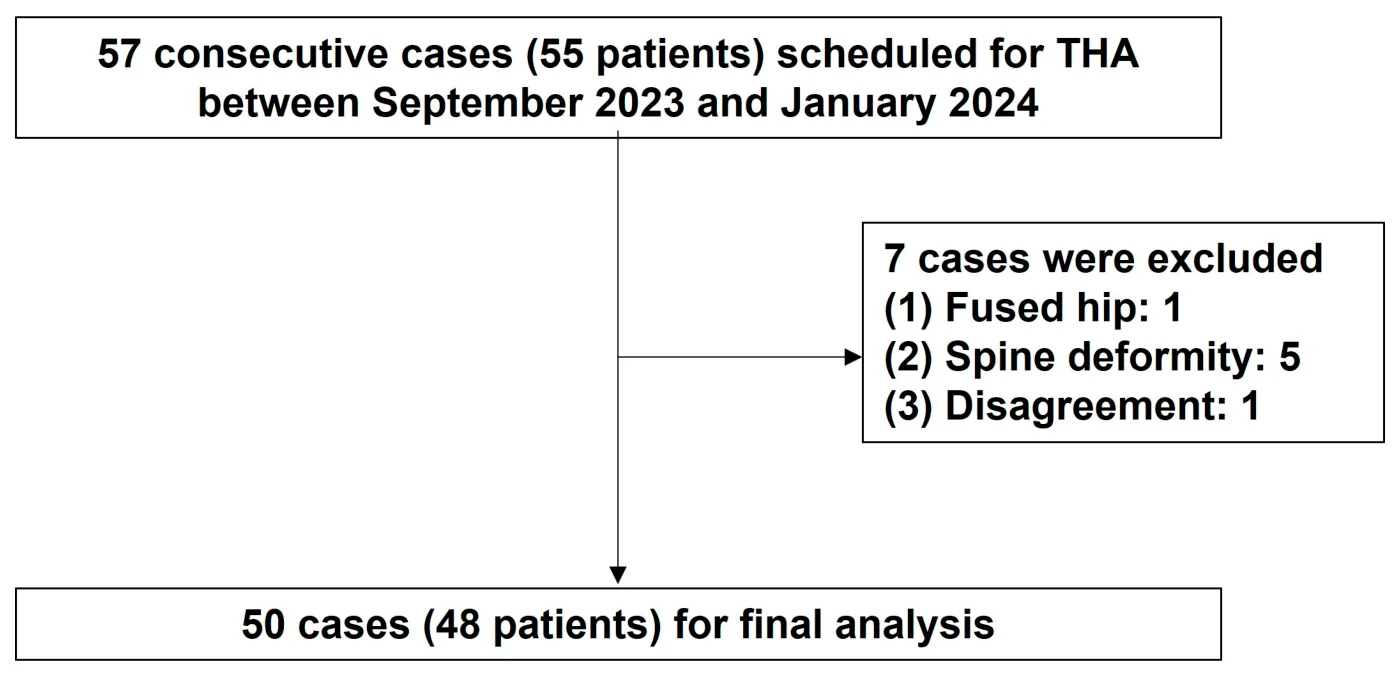
Flowchart of the study.

**Figure 3:**
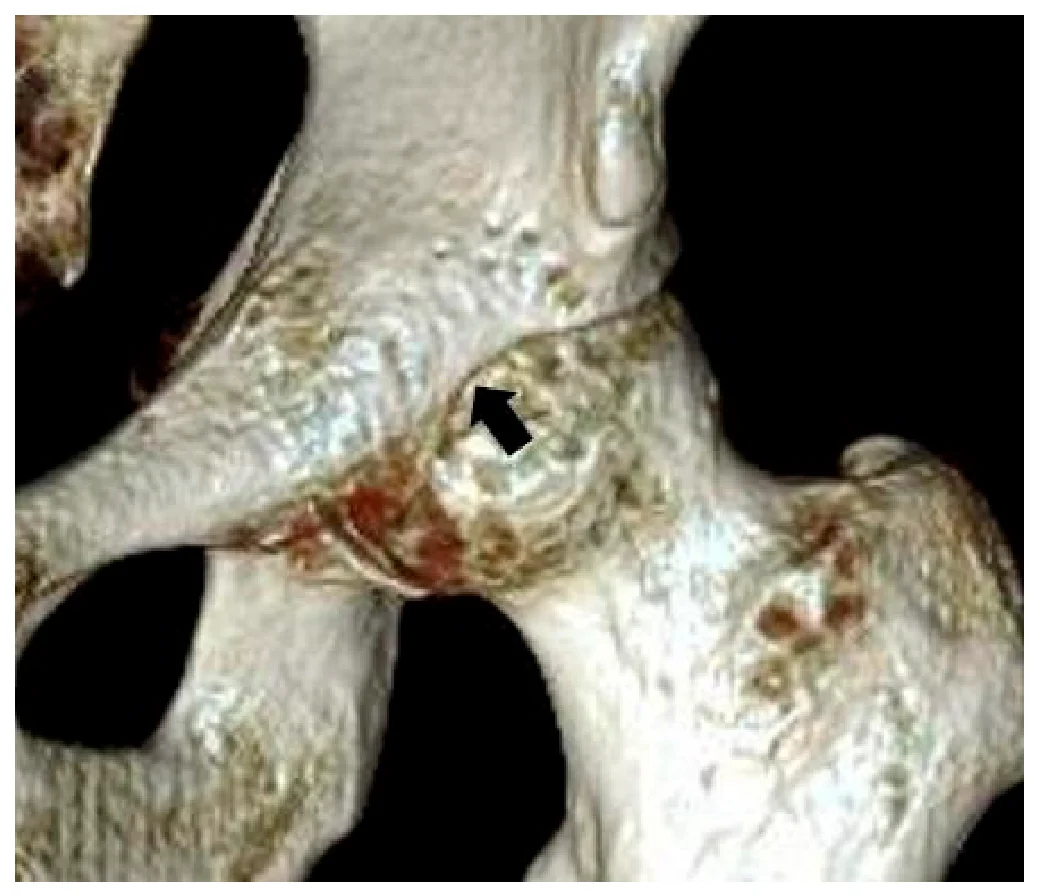
The anterior acetabular notch (AAN) at the anterior rim (black arrow) of the acetabulum.

Following institutional review board approval, 48 patients (50 hips) scheduled for primary THA between September 2023 and January 2024 at our tertiary hospital were prospectively enrolled. Of the 55 patients (57 hips) initially considered, seven cases were excluded according to the exclusion criteria. One case involved severe degenerative change in the hip, making it difficult to identify the bone margin in a fused hip. Five cases had spinal deformity (a difference of 10° or more for pelvic incidence minus lumbar lordosis) [17,18]. One patient refused to participate (Figure 2). Most patients were diagnosed with osteonecrosis (32 hips, 64%) or osteoarthritis (12 hips, 24%) (Table 1). Patients with spinal deformity were excluded because we intended to use the anterior acetabular notch (AAN) (Figure 3) as a bony landmark, as described by Ha et al. [14]. The AAN is close to the anterior pole of the acetabulum when the anterior pelvic plane (APP) is vertical; in spinal deformity, the APP is tilted forward or backward, so these cases were excluded.

### 2.2. Preoperative Planning and Geometric Model

**Figure 4:**
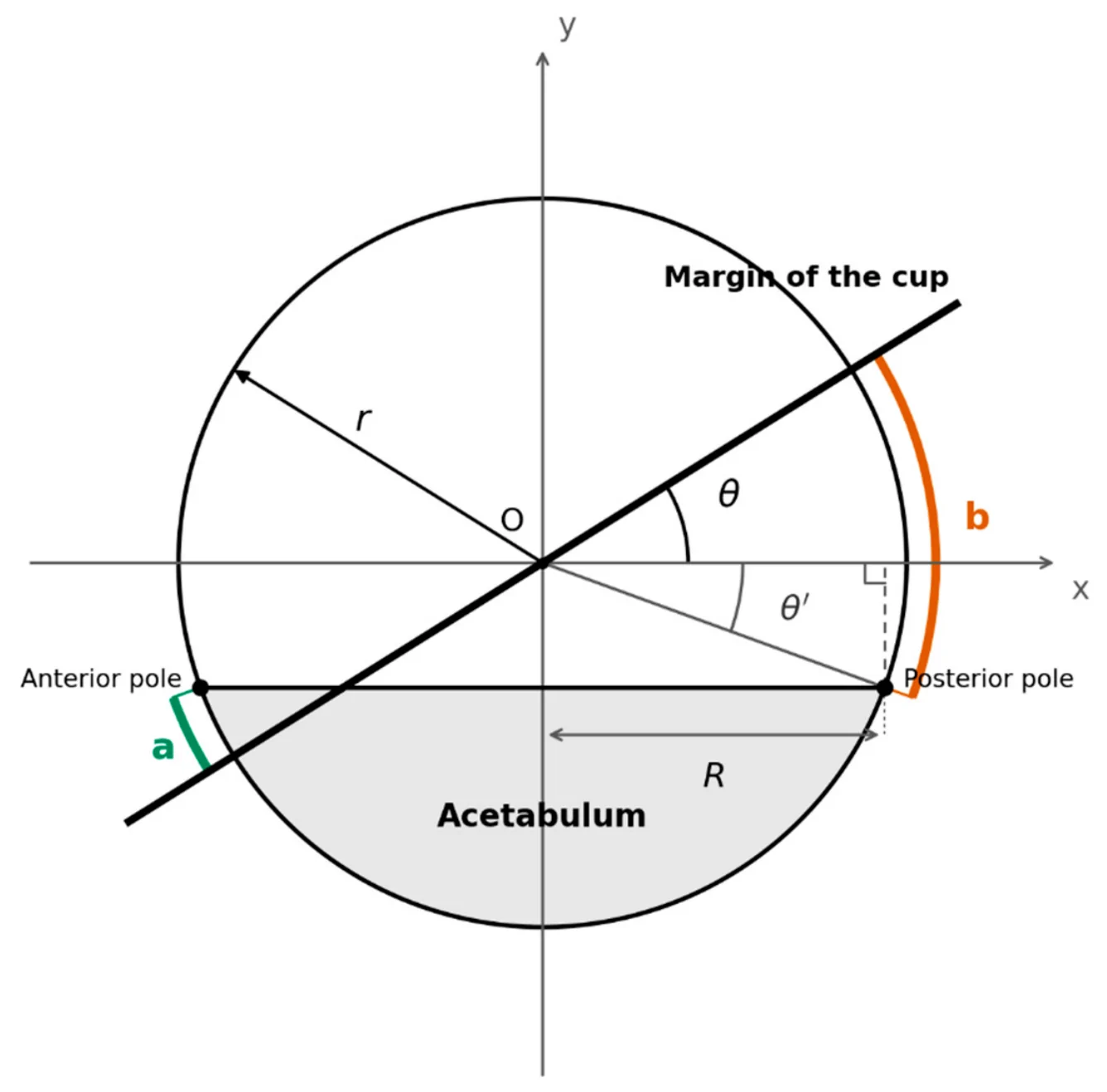
Schematic illustrating the geometric model for acetabular cup positioning. r, radius of the acetabular cup; θ, additional anteversion to be applied relative to the patient’s native anteversion; θ′, auxiliary angle between the x-axis and the line connecting O to each acetabular pole; R, half the distance between the anterior and posterior poles of the acetabulum; a and b, arc lengths measured along the cup rim from the anterior and posterior poles, respectively, to the cup margin.

We considered the AAN as the anterior pole and the point opposite the AAN as the posterior pole of the acetabulum. The posterior pole was determined with reference to the acetabular center. During preoperative planning, the axial CT image including the AAN was selected, and the posterior pole was defined as the point on the posterior acetabular rim located opposite the AAN across the acetabular center. The acetabular center was approximated from the femoral head and acetabular contour on the same axial image. Intraoperatively, after exposure of the acetabular rim, the posterior pole was identified as the point on the posterior rim farthest from the AAN, corresponding to the posterior rim point opposite the AAN through the acetabular center. As shown in Figure 4, if the values of r, θ (in radians), and R are known, the values of a and b can be calculated using the following equations based on circular measure. To make the geometry explicit, we defined a Cartesian coordinate system in the mid-axial CT plane, with its origin O at the center of the acetabular cup. The x-axis was aligned with the native interpolar axis, defined as the line connecting the anterior and posterior poles, and the y-axis was set perpendicular to this axis (Figure 4).

In this coordinate system, r denotes the radius of the acetabular cup determined during preoperative templating. R denotes half the distance between the anterior and posterior poles, such that the perpendicular projection of each pole onto the x-axis is located at a distance R from O. The angle θ represents the additional anteversion to be applied to the cup relative to the patient’s native anteversion. The angle θ′ is an auxiliary angle formed at O between the x-axis and the line connecting O to each pole. The quantities a and b represent the arc lengths measured along the cup rim from the anterior and posterior poles, respectively, to the cup margin.

All subsequent relationships are derived directly from circular geometry, in which arc length equals the radius multiplied by the central angle expressed in radians. Thus, θ and θ′ were expressed in radians when substituted into the arc-length equations. No small-angle or small-displacement approximation was used at any step. Therefore, the equations remain exact across the range of angular displacements encountered clinically. Although the illustration shows a shallow acetabulum, the same formulas apply to hemispherical or deeper configurations. First, the angle θ′ is defined:

(1) $$\theta^{\prime }\;=\;{cos}^{-1}(\frac{R}{r})$$

The lengths of segments a and b are then calculated as follows:

(2) $$a\;=\;r\theta \;-\;r\theta^{\prime }\;=\;r\theta \;-\;r{cos}^{-1}(\frac{R}{r})$$

(3) $$b=r\theta +r\theta^{\prime }=r\theta +r{cos}^{-1}(\frac{R}{r})$$

**Figure 5:**
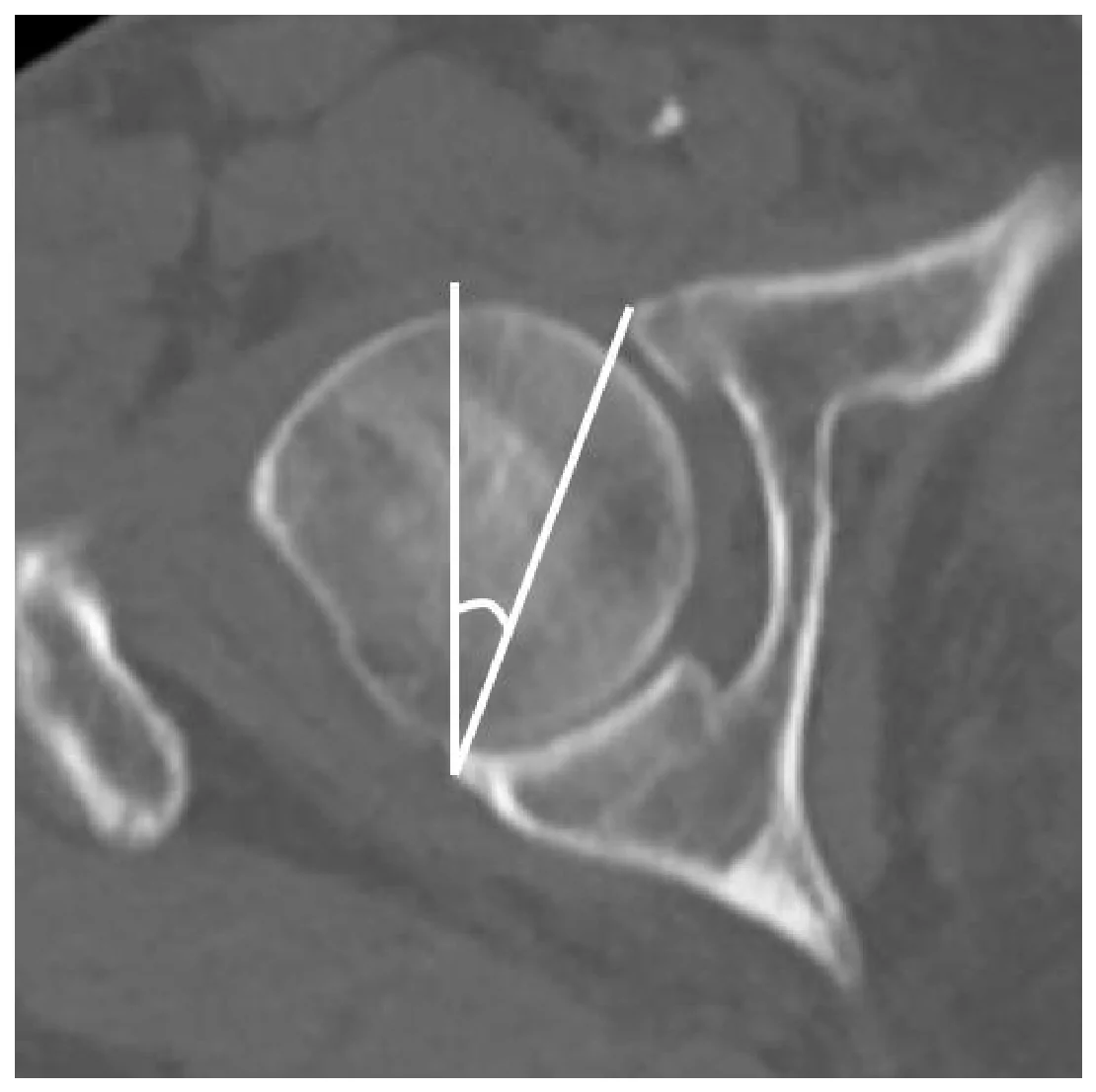
Acetabular anteversion measured on a mid-axial CT image.

Native acetabular anteversion was measured on CT as the angle between a line perpendicular to the line connecting the centers of the two femoral heads and a line connecting the anterior and posterior bony margins of the acetabulum on the largest cross-section of the femoral head in the axial plane (Figure 5). The target was based on the Lewinnek safe zone of 15° ± 10° [3]. However, the anteversion measured on CT is anatomical anteversion as defined by Murray, whereas the Lewinnek safe zone is based on radiographic anteversion [3,19]. Therefore, to set an anatomical anteversion target corresponding to a radiographic anteversion of 15° and a radiographic inclination of 40°, we applied Murray’s formula [19,20]:

(4) $$Anatomical\;anteversion\;=\;{tan}^{-1}(\frac{tan(radiographic\;anteversion)}{sin(radiographic\;inclination)})$$

**Figure 6:**
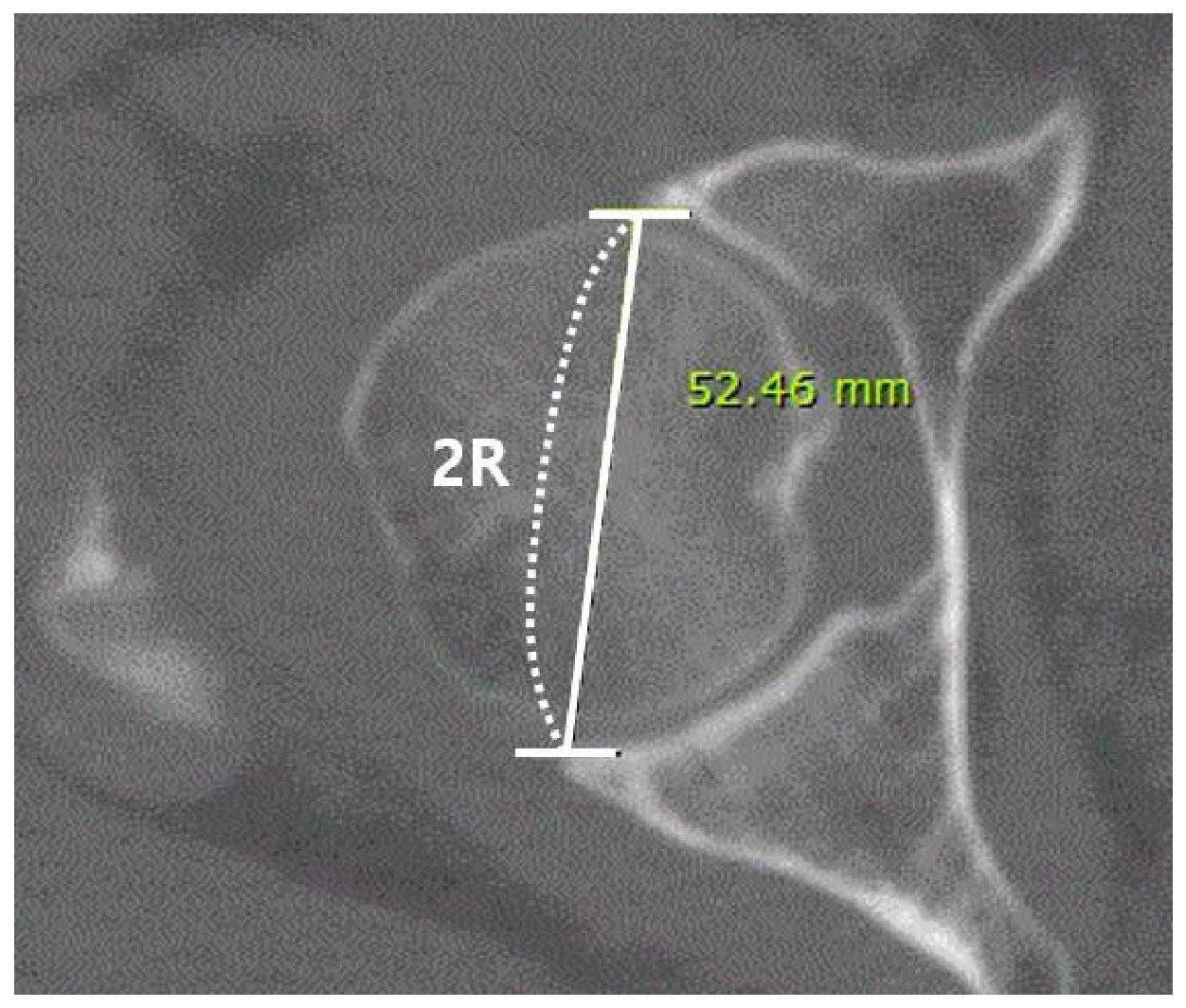
The distance between the anterior and posterior poles of the acetabulum measured on a mid-axial CT image.

This yielded approximately 22.6°, which we rounded to 23° for simplicity. Thus, θ equals 23° minus the patient’s anteversion. Because the arc-length relationships in Equations (2) and (3) require θ in radians, this angular value (in degrees) was converted to radians before being substituted into the equations. Finally, R is half the distance between the anterior and posterior poles of the acetabulum. Although direct intraoperative measurement would be most accurate, R was measured preoperatively using picture archiving and communication system (PACS) software (INFINITT PACS M6; INFINITT Healthcare, Seoul, Republic of Korea) to avoid surgical delay (Figure 6). Using r, θ, and R, we determined a and b preoperatively. Because acetabular cups of different sizes might be needed, a and b were also pre-calculated for one size larger and one size smaller.

### 2.3. Surgical Technique

**Figure 7:**
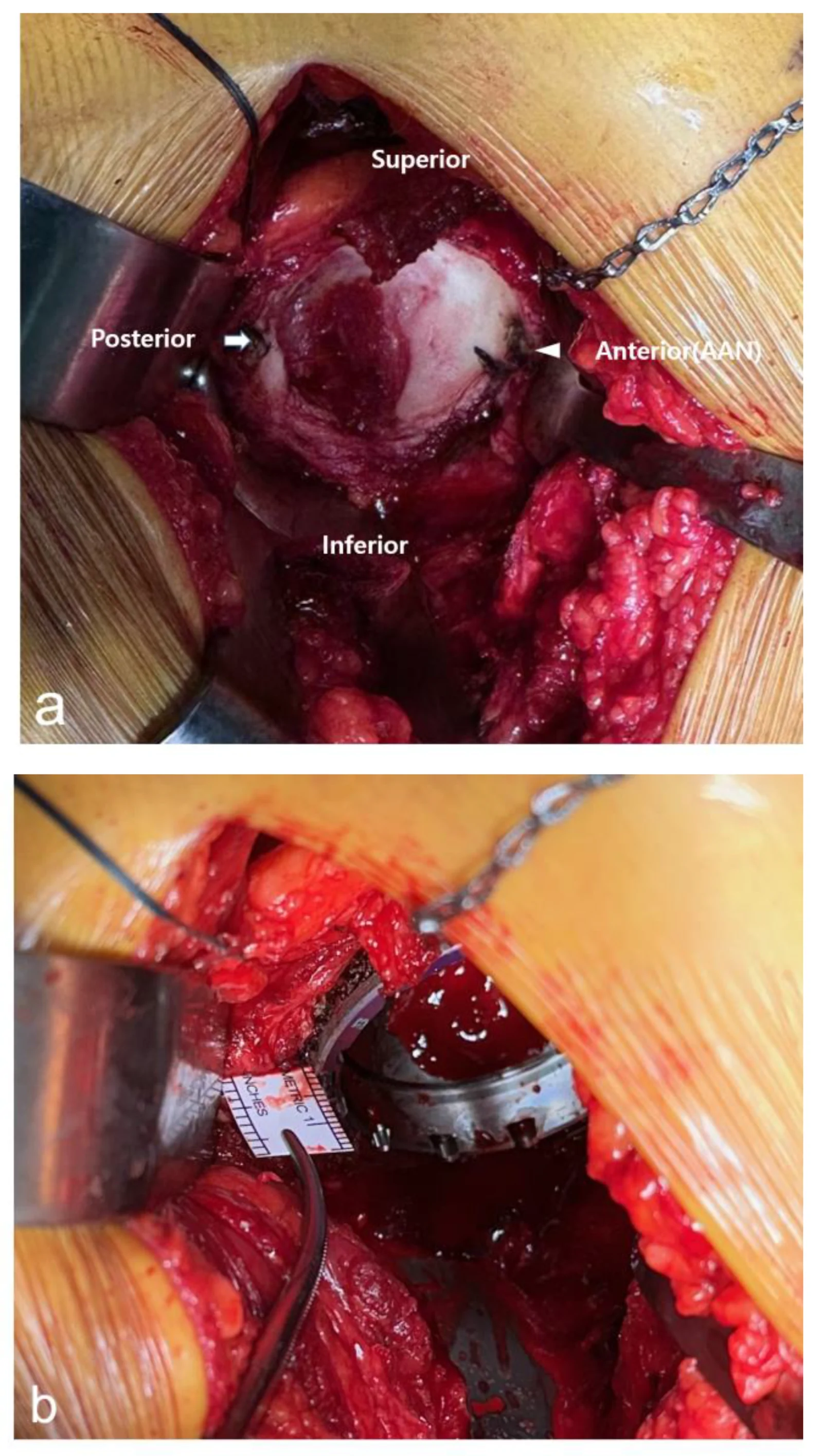
(**a**) After exposing the bony margin, the anterior and posterior poles were marked. (**b**) The distance between each pole and the cup margin was measured to confirm that it matched the calculated a and b.

All surgeries were performed by a single high-volume arthroplasty surgeon using a piriformis-sparing posterolateral approach. After the acetabular labrum was completely removed to expose the bony margin, the AAN and the posterior pole were identified and marked (Figure 7a). The acetabulum was reamed until bleeding from the subchondral bone bed was exposed, while the subchondral bone plate was preserved as much as possible. The intended cup height was determined during preoperative templating with reference to the native acetabular center and expected host-bone coverage. Intraoperatively, this planned level was reproduced by sequential reaming along the native acetabular floor. When medialization was performed for a shallow acetabulum, it was limited so that the anterior and posterior poles were not violated, provided that coverage was sufficient. Cementless acetabular components (G7; Zimmer Biomet, Warsaw, IN, USA; and Mirabo; Corentec, Seoul, Republic of Korea) were used in all cases (Table 1). After the acetabular component was positioned, the distance between each pole and the cup margin was measured to confirm that it matched the calculated a and b (Figure 7b).

### 2.4. Postoperative Evaluation

On the fifth postoperative day, CT was performed, and anteversion was measured to determine whether it approximated the target of 23°. Postoperative radiographic inclination was also measured to calculate radiographic anteversion. Postoperative CT-measured anatomical anteversion and radiographic inclination were used to calculate radiographic anteversion with the following formula [19,20], to determine whether the cup was within the Lewinnek safe zone:

(5) $$Radiographic\;anteversion\;=\;{tan}^{-1}(tan(anatomical\;anteversion)\;\times \;sin(radiographic\;inclination))$$

The proportion of outliers from the Lewinnek safe zone was evaluated using the calculated radiographic anteversion. Clinically, the incidence of dislocation and the mHHS were assessed 3 months postoperatively.

### 2.5. Statistical Analysis

Summary data are expressed as means ± standard deviations for continuous variables and as numbers and frequencies (%) for categorical variables. The positioning error was calculated by comparing the postoperative CT-measured anatomical anteversion with the predefined target of 23°. The signed error was defined as the postoperative CT anteversion minus the target, and the absolute error as the absolute value of this difference. The corresponding percentage errors were calculated as the signed or absolute error divided by the 23° target and multiplied by 100 and were averaged across all hips. In addition, the systematic difference (bias) was assessed as the mean signed difference between the measured and target anteversion. Because this was a prospective proof-of-concept evaluation of a new positioning technique, the sample size was chosen to estimate the safe-zone outlier rate with reasonable precision rather than to power a comparison against a control group. For a technique intended to reduce safe-zone outliers, observing no outliers in 50 hips gives, by the rule of three, an approximate upper bound of the 95% confidence interval for the true outlier rate of 6% (3/50). This was considered acceptable for evaluating the feasibility and accuracy of the method in a proof-of-concept setting.

## 3. Results

**Figure 8:**
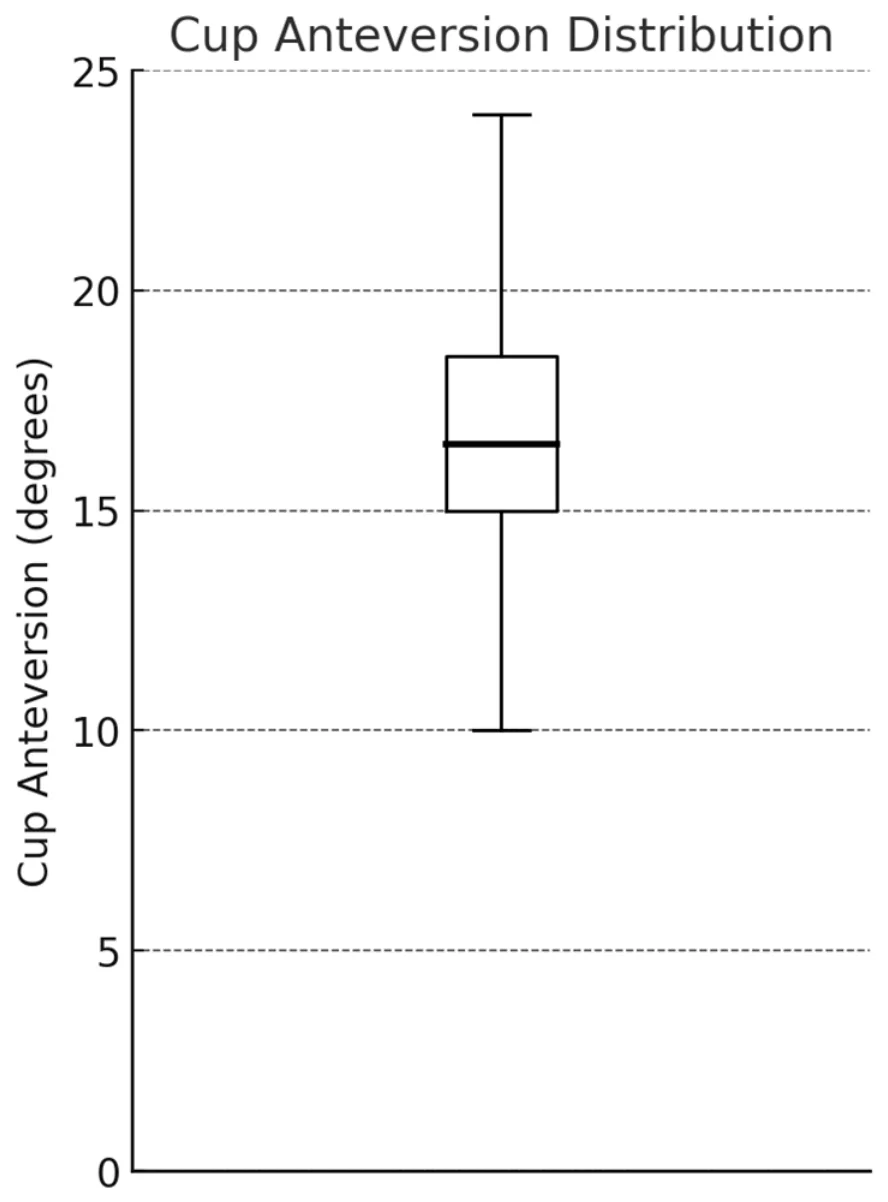
Box plot illustrating the distribution of calculated radiographic cup anteversion angles.

Most patients were diagnosed with osteonecrosis (32 hips, 64%) or osteoarthritis (12 hips, 24%). Cementless acetabular components were used in all cases (Table 1). The mean postoperative CT anteversion of the acetabular cup was 25.1° (range, 16.0–35.0°). The mean signed error relative to the 23° target was +2.1° ± 4.9° (range, −7.0° to +12.0°; mean signed percentage error, +9.2% ± 21.2%; range, −30.4% to +52.2%). The mean absolute error was 4.0° ± 3.5° (range, 0–12.0°; mean absolute percentage error, 17.4% ± 15.0%; range, 0–52.2%). The mean postoperative radiographic inclination was 39.9° (range, 33.2–44.7°). The radiographic anteversion calculated from the CT anteversion of the acetabular cup was 16.8° (range, 10.0–24.0°) (Figure 8), and all hips were within the Lewinnek safe zone. The mean mHHS at 3 months postoperatively was 92.4. No dislocations were observed at 3 months postoperatively (Table 2).

## 4. Discussion

In this study, we placed all acetabular components within the Lewinnek safe zone by applying a cup-positioning technique based on preoperative CT assessment and an understanding of acetabular bony landmarks.

Several studies have shown that robotic surgery improves the precision of acetabular cup placement; however, this approach is associated with high cost and a learning curve [7,8,9]. Domb et al. reported minimum 5-year outcomes in 66 patients who underwent robotic-assisted primary THA (rTHA) and showed that 97.0% of the acetabular components were within the Lewinnek safe zone [21]. Kong et al. reviewed the first 100 robot-assisted THAs performed by an experienced surgeon and suggested that a learning curve of 14 cases was required to become proficient in rTHA, with 92% of cases within the Lewinnek safe zone [22]. Kayani et al. evaluated 50 patients who underwent robotic-arm-assisted acetabular cup positioning during THA and found a learning curve of 12 cases in terms of operative time; no outliers from the Lewinnek safe zone were identified [23] (Table 3).

In our study, the anteversion of the acetabular components had a mean error of 4°, indicating successful results. When expressed as radiographic anteversion, all 50 hips were within the Lewinnek safe zone. No dislocations were observed at the 3-month follow-up, and excellent clinical outcomes were confirmed by the mHHS. Recent studies have suggested that navigation and robotic surgery can position the acetabular cup more accurately; however, we found that understanding the patient’s bony landmarks and predicting the cup position achieved an outlier rate comparable to those reported in previous navigation or robotic studies, although direct comparison was not possible because this study did not include a control group. During surgery, changes in patient position can influence pelvic position, which can in turn affect the orientation of the acetabular cup [24,25,26]. However, intraoperative bony landmarks are patient-specific and independent of patient position. A further advantage is that the technique replaces intraoperative visual estimation of version—the principal source of inter-surgeon variability in freehand cup placement—with patient-specific, CT-derived target distances, which may improve the consistency of cup positioning.

This study had several limitations. First, all procedures were performed by a single experienced surgeon at a single institution, so the reproducibility of the technique among surgeons with different levels of experience remains unknown; multi-surgeon, multicenter validation is therefore required. Second, this proof-of-concept study did not include a control group; therefore, superiority over conventional freehand, navigation, or robotic-assisted techniques cannot be concluded. Third, the study population was selected to test the feasibility of the geometric model in standard primary THA. Patients with severe deformity, fused hips, unclear bony landmarks, and major spinopelvic abnormalities were excluded, limiting generalizability to complex cases. Fourth, preoperative CT is required, which involves additional radiation exposure and cost but enables assessment of the acetabular rim. Fifth, the method assumes that the acetabulum approximates part of a sphere and that the cup is implanted near the planned acetabular level. Changes in cup height or medialization may alter the relationship between the cup rim and acetabular rim, making a and b less accurate; therefore, caution is required in dysplastic hips or cases requiring substantial medialization or superior cup placement. Sixth, using the AAN as the anterior pole may be affected by sagittal pelvic alignment. The mean measured anteversion exceeded the target by 2.1°, suggesting a small systematic overshoot, and future refinements may need to incorporate pelvic tilt. Seventh, intraoperative measurement of a and b using graph paper was approximate and may have been affected by parallax error, limited visualization, and difficulty aligning the scale with the curved acetabular rim. Therefore, it should be regarded as a practical intraoperative guide rather than a precise quantitative measurement. Finally, the 3-month follow-up is insufficient to fully evaluate long-term clinical outcomes or late complications; thus, the absence of dislocation and favorable mHHS should be interpreted as early clinical findings.

## 5. Conclusions

In this prospective proof-of-concept study, acetabular components were positioned within the Lewinnek safe zone using CT-based acetabular bony landmarks in 48 patients (50 hips) undergoing standard primary THA. These findings support the feasibility and accuracy of the proposed technique in this cohort; however, further controlled studies involving multiple surgeons, longer follow-up, and more complex cases are required to confirm its reproducibility and clinical advantage.

## Figures and Tables

**Table 1:** Patient demographics.

Demographics	Value
Number	50
Age, mean ± SD, years	58.9 ± 13.7
Sex	
Female	23 (46%)
Male	27 (54%)
BMI, mean ± SD, kg/m^2^	24.4 ± 3.5
Cause of THA	
Osteonecrosis	32 (64%)
Osteoarthritis	12 (24%)
Femoral neck fracture	4 (8%)
Femoral head insufficiency fracture	1 (2%)
Rheumatoid arthritis	1 (2%)
Laterality	
Right	28 (56%)
Left	22 (44%)
Surgical approach (posterolateral)	50 (100%)
Preoperative acetabular anteversion, mean ± SD, °	17.5 ± 5.4
Acetabular component	
G7 (Zimmer Biomet)	46 (92%)
Mirabo (Corentec)	4 (8%)
Bearing liner	
E1 fixed (Zimmer Biomet)	30 (60%)
E1 dual mobility (Zimmer Biomet)	18 (36%)
Ceramic (Biolox Delta, CeramTec)	2 (4%)

BMI, body mass index; SD, standard deviation; THA, total hip arthroplasty.

**Table 2:** Postoperative outcomes.

Parameter	Value
CT anteversion, mean ± SD	25.1° ± 4.9
Signed error from target anteversion, mean ± SD	2.1° ± 4.9
Absolute error from target anteversion, mean ± SD	4.0° ± 3.5
Radiographic inclination, mean ± SD	39.9° ± 2.2
Calculated radiographic anteversion, mean ± SD	16.8° ± 3.5
Dislocation, n	0
mHHS, mean ± SD	92.4 ± 9.1

CT, computed tomography; mHHS, modified Harris hip score; SD, standard deviation.

**Table 3:** Studies on acetabular component positioning.

Author	Year	Method	No. of Hips	Cup Anteversion (°)	Outliers (%)
Domb et al. [21]	2020	rTHA	66	18.4	3
Kong et al. [22]	2020	rTHA	86	19.1	8.1
Kayani et al. [23]	2021	rTHA	50	NR	0
Current study	2024	Anatomical landmarks	50	16.8	0

NR, not reported; rTHA, robotic-arm-assisted total hip arthroplasty.

## Data Availability

The data presented in this study are available on request from the corresponding author.
